# Mechanistic Involvement of Long Non-Coding RNAs in Oncotherapeutics Resistance in Triple-Negative Breast Cancer

**DOI:** 10.3390/cells9061511

**Published:** 2020-06-21

**Authors:** Samarth Kansara, Vijay Pandey, Peter E. Lobie, Gautam Sethi, Manoj Garg, Amit Kumar Pandey

**Affiliations:** 1 Amity Institute of Biotechnology, Amity University Haryana, Panchgaon, Manesar, Haryana 122413, India; kansara.manojkumar@student.amity.edu; 2 Tsinghua-Berkeley Shenzhen Institute, Tsinghua University, Shenzhen 518005, China; vijay.pandey@sz.tsinghua.edu.cn (V.P.); pelobie@sz.tsinghua.edu.cn (P.E.L.); 3 Shenzhen Bay Laboratory, Shenzhen 518055, China; 4 Department of Pharmacology, Yong Loo Lin School of Medicine, National University of Singapore, Singapore 117600, Singapore; 5 Amity Institute of Molecular Medicine and Stem Cell Research (AIMMSCR), Amity University, Sector-125, Noida 201313, India; mgarg@amity.edu

**Keywords:** long non-coding RNA (lncRNA), triple-negative breast cancer (TNBC), chemoresistance, acquired drug resistance, oncotherapeutics

## Abstract

Triple-negative breast cancer (TNBC) is one of the most lethal forms of breast cancer (BC), with a significant disease burden worldwide. Chemoresistance and lack of targeted therapeutics are major hindrances to effective treatments in the clinic and are crucial causes of a worse prognosis and high rate of relapse/recurrence in patients diagnosed with TNBC. In the last decade, long non-coding RNAs (lncRNAs) have been found to perform a pivotal role in most cellular functions. The aberrant functional expression of lncRNAs plays an ever-increasing role in the progression of diverse malignancies, including TNBC. Therefore, lncRNAs have been recently studied as predictors and modifiers of chemoresistance. Our review discusses the potential involvement of lncRNAs in drug-resistant mechanisms commonly found in TNBC and highlights various therapeutic strategies to target lncRNAs in this malignancy.

## 1. Introduction

Breast cancer (BC) is the most diagnosed cancer in women worldwide and is significantly associated with cancer-related mortality [1]. Breast carcinoma is very heterogeneous and is subcategorized based on molecular and genomic aberrations into luminal A and B, human epidermal growth factor receptor 2 (HER2), and basal subtypes. Patients with luminal A and B are positive for estrogen receptor (ER) and progesterone receptors (PR) and exhibit a good prognosis. Ki-67 expression is low in luminal A, which also lacks HER2 expression. The luminal B subtype exhibits high Ki-67 expression and is HER-2-positive. The HER-2 subtypes are characterized by lack of ER and PR expressions, HER-2 positivity, high Ki-67 expression, and exhibit a poor prognosis [2]. An aggressive basal (clinical) subtype is triple-negative BC (TNBC), defined by a lack of expression of ER, PR, and HER-2, with a prominent basal marker expression, resulting in a high recurrence rate [3,4]. TNBC accounts for 15% to 20% of BC [5,6,7]. TNBC is also common amongst women in developing nations, with an occurrence rate of between 20% to 43% [8]. The Lehmann–Pietenpol classification system divides TNBC into another six subtypes based on distinct gene expression patterns [9,10].

Surgery remains the first line of treatment for TNBC, followed by cytotoxic chemotherapy, which includes anthracyclines, cisplatin, and microtubule-binding agents, in addition to radiotherapy [11,12]. A significant number of patients display resistance to these treatments, associated with a high rate of metastasis, faster relapse of the disease, and worse survival outcome than other breast cancer subtypes [13]. Such acquired multidrug resistance (MDR) is due to the increased expression of the ATP-binding cassette (ABC) transporter superfamily. The ABC family includes the MDR-associated transporter P-glycoprotein (MDR-1) or proteins from the MDR-associated protein (MRP) family [14].

Furthermore, the pharmacokinetic features of the utilized chemotherapeutic agents are poor, with undesirable side effects such as myelo- and immunosuppression, cardiotoxicity, fluid retention, and leukopenia [15,16]. Thus, there is an urgent need for the identification of safe, novel, and effective therapeutic options for TNBC. To attain this goal, there is an urgent requirement for the exploration of actionable mechanisms of chemoresistance in TNBC. Recent advances in high-throughput genomic and transcriptomic RNA sequencing technologies and the completion of the Encyclopedia of DNA Elements (ENCODE) project showed that about 70% of the total human genome bases are part of the transcript but only 2% of the genome is translated into proteins [17]. Earlier, these nontranslated parts, known as non-coding RNAs (ncRNAs), were categorized as transcriptional noise. Several groups have shown that ncRNAs play an important role in multiple biological processes, such as imprinting; splicing; RNA decay; differentiation; and human diseases such as diabetes, cardiovascular disease, and cancers. These ncRNAs can be divided into two classes on the basis of their size and function: (1) short ncRNAs that are less than 200-nucleotides long and include microRNAs (miRNAs), small interfering RNAs (siRNAs), small nucleolar RNAs (snoRNAs), and Piwi-interacting RNAs (piRNA) and (2) long non-coding RNAs (lncRNAs) are more than 200-nucleotides long and are characterized by transcription through RNA polymerase II, a 5′ cap, a transcription start site, and polyadenylation [18].

## 2. LncRNAs

The diverse regulatory functions of lncRNAs in various biological processes [19,20] control the expression of genes by different mechanisms [21]. These mechanisms include acting as decoys (molecular sinks for transcription factors and repressors to facilitate gene activation and silencing), guides (bind to enzymatically active or regulatory protein complexes and lead them to a specific target), scaffolds (act as a central platform to which various protein complexes can tether and lead to specific genomic locations), signaling molecules (associated with specific signaling pathways), and miRNA sponges (modulating miRNA expression) [22]. Interestingly, lncRNAs can be transcribed from gene regulatory regions (enhancers and promoters), intergenic regions, introns, antisense strands, untranslated regions (UTR), and telomeres [23,24]. LncRNAs can form complexes with DNA, RNA, and proteins to perform their specific activities [25].

### 2.1. Mechanisms of a Function of LncRNAs

**Figure 1:**
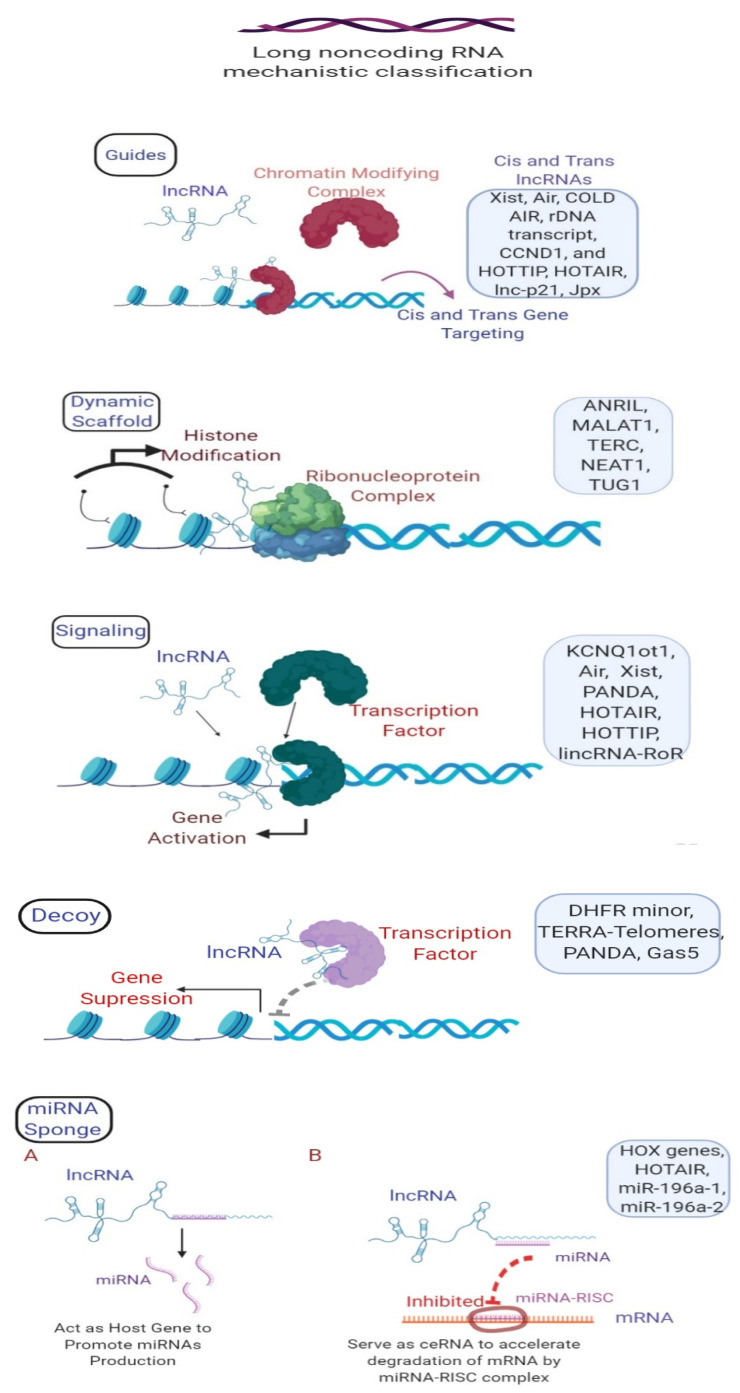
Long non-coding RNA mechanistic classification. **Guides**—long non-coding lncRNA interacts with enzymatically active or modifying complexes and directs them to a specific target. **Dynamic Scaffolds**—lncRNA serves as a central platform for multiple protein complexes and/or other cofactors directed to the specific genomic location. **Signaling molecules**—lncRNAs associated with specific signaling molecules for the activation of genetic and molecular pathways. **Decoys**—lncRNAs can bind to transcription factors or repressors to regulate gene activation and silencing. **Micro (mi)RNA sponges**—lncRNAs modulate miRNA expression; (**A**) they act as a host gene to promote miRNA production, and (**B**) they serve as competitive endogenous RNA (ceRNA) to accelerate the degradation of mRNA (messenger RNA) by the miRNA–RISC (RNA-induced silencing complex) complex. (Figure made using BioRender (https://biorender.com/)).

Based on the mechanisms, lncRNAs are classified into guides, scaffolds, signaling molecules, decoys, and miRNA sponges (Figure 1).

#### 2.1.1. Guide lncRNAs

Expression of cis (neighboring) or trans (distantly located) genes is guided by lncRNAs in a manner that cannot be predicted based on the sequence of lncRNA. Xist (X-inactive specific transcript), Air, HOTTIP (HOXA Distal Transcript Antisense RNA), Cold Assisted Intronic Noncoding RNA (COLD AIR), cyclin D1 (CCND1), and rDNA transcripts are well-studied lncRNAs that are cis-acting guides. The X inactivation center (XIC), where lncRNAs regulate genes through cis action, is the most well-understood [26]. Reports have shown that some lncRNAs, such as Homeobox (HOX) transcript antisense intergenic RNA (HOTAIR), lnc-p21, Just proximal to Xist (Jpx), and other PRC2-bound RNAs, regulate expressions in a trans manner [27]. The upregulation of HOTAIR (Hox lncRNA) has been shown to increase tumor growth, as well as chemoresistance through the Wnt/β-catenin pathway in colon cancer [28]. X-inactive specific transcript (Xist), a cis-acting guide lncRNA, downregulates in TNBC, which is associated with upregulated miR-454. The knockdown of miR-454 regulates Xist expression and inhibits cell proliferation, epithelial mesenchymal transition (EMT), and induced cell apoptosis in TNBC [29]. So, Xist serves as a model example for studying deeper guide lncRNA and their mechanisms of action. Moreover, the expression of guide lncRNA HOTTIP is found considerably associated with the lymph node status, tumor size, and overall survival in breast cancer patients [30].

#### 2.1.2. Scaffold lncRNAs

Scaffold lncRNA (Antisense non-coding RNA at the INK4 locus)ANRIL—PRC2 and PRC1 are antisense lncRNAs that originate from the INK4b/ ARF/INK4a locus and are vital for expression of the cis protein-coding genes. The interaction of ANRIL with PRC1 and PRC2 leads to transcriptional repression of the target INK4b locus [31]. LncRNA GClnc1 (gastric cancer-associated lncRNA 1), which has been shown to promote gastric cancer, specifies the pattern of histone modification on superoxide dismutase 2 through a modular scaffold of WDR5 and KAT2A complexes [32]. lncRNAs also act as scaffolds by binding to DNA in order to introduce a protein into the locus of the gene. For example, lncRNA HOTAIR in breast cancer functions as a scaffold to target and create a bridge between the PRC2 complex and the LSD1 H3K1 demethylase complex and recruits both in order to uniformly alter several histone modifications. This histone modification alters the expression pattern and increases breast cancer invasiveness and metastasis. This suggests that lncRNA provides a binding surface to assemble selected histone modification enzymes [33].

#### 2.1.3. Signaling lncRNAs

Signaling lncRNAs, including KCNQ1ot1, Air, and Xist, have been demonstrated to regulate allelic specificity. Imprinting is an epigenetic regulatory mechanism that restricts either maternal or paternal alleles out of the two autosomal genes contributed by each parent. LncRNAs Kcnq1ot1 and Air, which map to Kcnq1 and Igf2r in mouse placenta, accumulate at the promoter of silenced alleles and intervene with repressive histone alterations in an allele-specific manner [34]. HOTAIR and HOTTIP lncRNA also mediate anatomic-specific expressions. Furthermore, linc-p21 lncRNA, which is located upstream of the *CDKN1A* gene, regulates p53-mediated gene regulation on DNA damage and subsequent apoptosis. Another lncRNA, PANDAR(p21-associated ncRNA DNA damage-activated), located upstream of *CDKN1A*, is activated after DNA damage, wherein TP53 interacts with CDKN1A. PANDAR, on interacting with the transcription factor NF-YA, then facilitates cell cycle arrest and terminates proapoptotic gene expression [35]. OCT4, SOX2, and NANOG also colocalize to the promoter of LncRNA-ROR and regulate reprogramming and pluripotency [36]. LncRNA BC antiestrogen resistance 4 (BCAR4) has been observed with the metastasis of TNBC through its involvement in the Hippo and Hedgehog signaling to modulate glucose metabolism and act as a potential therapeutic target for TNBC treatment [37].

#### 2.1.4. Decoy lncRNAs

Recently, lncRNAs have been shown to inhibit the protein function by acting as a decoy molecule, where they bind and sequester specific protein sequences or microRNA away from their native targets [38]. These decoy lncRNAs act as a competitive inhibitor, thereby modulating the protein function and regulating gene expression. lncRNAs, such as Gas5 (growth arrest-specific 5), act as a molecular decoy by forming an RNA motif from one of its stem-loop structures, thereby repressing the glucocorticoid receptor and regulating the metabolic activities and cell survival during starvation [39]. MEG3 (Maternally expressed 3) lncRNA has been reported to act as a molecular decoy for tumor-related microRNAs such as miR-421. The downregulation of MEG3 linked with lymph node metastasis in HER-2-positive BC, and upregulation leads to reduced cell proliferation via the sequestration of miR-421 by negatively regulating E-cadherin expression [40].

#### 2.1.5. miRNA Sponges

LncRNA-based modulation of the expression of miRNAs critically contributes in the progression and metastasis of various human malignancies [41]. During metastasis, it has been observed that lncRNA HOTAIR indirectly inhibits miR-7 [42]. The inhibition of miR-7 by HOTAIR plays a vital role in the suppression of breast cancer stem cell functions by altering the expression of an oncogene, the SETDB1 function. Moreover, the miR-7-based downregulation of SETDB1 resulted in suppression of the STAT-3 pathway and led to inactivation of epithelial-to-mesenchymal transition-related genes in BC stem-like cells [42]. Interestingly, the silencing of HOTAIR lncRNAs resulted in the suppression of HoxD10, which, in turn, induced the expression of miR-7 and inhibited the metastasis of TNBC [43].

Therefore, lncRNAs-based expression effects regulate diverse cellular processes, such as proliferation, epigenetic regulation, migration, angiogenesis, cell-cycle control, metastasis, and apoptosis, and lncRNAs are linked to several disease states, including cancer [44].

### 2.2. LncRNAs Involved in TNBC

**Figure 2:**
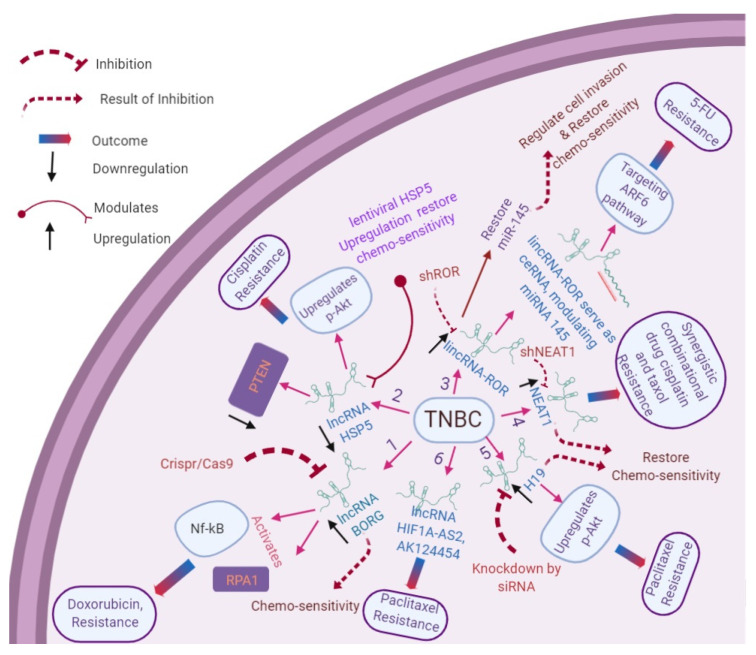
Role of lncRNAs in the drug resistance of triple-negative breast cancer (TNBC). (1) LncRNA BORG (BMP/OP-Responsive Gene) activates NF-ĸB [56], and RPA1 signaling is responsible for doxorubicin resistance; the modulating BORG expression restores chemosensitivity in TNBC. (2) LncRNA HSP5 downregulates PTEN and upregulates p-AKT expression, which is directly responsible for cisplatin resistance in TNBC, whereas the restoration of HSP5 expression leads to the reestablishment of drug sensitivity in TNBC. (3) LncRNA-ROR serves as a ceRNA, where the upregulation of ROR leads to the downregulation of miR-145 via the ARF6 pathway, which is responsible for 5-fluorouracil (FU) resistance and metastasis in TNBC. The knockdown of ROR by using shROR leads to the restoration of drug sensitivity and regulation of TNBC invasion. (4) The upregulation of NEAT1 in TNBC is responsible for the synergistic and combinational drug resistance of cisplatin and taxol. The knockdown of NEAT1 by shNEAT1 leads to sensitization of the cell to chemotherapy. (5) The upregulation of lncRNA H19 regulates the AKT signaling pathway responsible for paclitaxel resistance. The knockdown of H19 restores chemosensitivity in TNBC. (6) LncRNA HIF1A-AS2 and AK124454 serve as integrated mRNA-lncRNA signatures responsible for paclitaxel resistance in TNBC. (Figure made using *BioRender*).

The association of lncRNAs with TNBC is summarized in Figure 2. LncRNAs modulate the expression of other lncRNAs, such as lncRNA H19 (H19 Imprinted Maternally Expressed Transcript), which functions as an oncogene in TNBC and estrogen-sensitive BC and promotes cell cycle progression by interacting with E2F1. The expression of H19 is negatively associated with lncRNA PTCSC3 (Papillary thyroid carcinoma susceptibility candidate 3) in TNBC. LncRNA PTCSC3 prevents TNBC cell proliferation by downregulating lncRNA H19 [45]. It has been shown that PTCSC3 is involved in STAT3 [46] and WNT signaling pathways [47,48,49]. Thus, crosstalk between WNT and STAT3 signaling pathways is mediated by lncRNAs H19 and PTCS3. Moreover, lncRNAs such as MIR100HG establish an RNA–DNA triplex structure, promote cell proliferation, and regulate the *CDKN1B* gene to control the cell cycle in TNBC. A reduced expression of MIR100HG results in cell cycle arrest at the G1 phase and a reduction in cell proliferation [50]. The classical WNT signaling pathway plays an essential role in regulating various cellular processes, such as cell migration, invasion, proliferation, differentiation, and cell apoptosis [51]. WNT signaling regulators contribute to TNBC progression through the lipoprotein receptor-related protein 6 (LRP6) coreceptor, the Frizzled (FZD) family receptors, and the ROR receptor [52]. lncRNA AWPPH (lncRNA associated with poor prognosis of HCC) promotes tumor growth in TNBC by upregulating the FZD7 receptor [53]. Various lncRNAs such as LINP1(LncRNA In Non-Homologous End Joining Pathway 1) are regulated by TP53 and the epidermal growth factor receptor (EGFR), which is overexpressed in TNBC and regulates the double-strand DNA break repair by the nonhomologous end-joining (NHEJ) pathway. The downregulation of LINP1 can enhance the sensitivity of TNBC against radiotherapy [54]. With the improvements in computational approaches and high-throughput RNA sequencing, a large proportion of lncRNAs have been identified. However, their expression profiles, mechanisms, and associated functions in the development and progression of TNBC broadly remain unclear [55].

## 3. Mechanisms Related to Oncotherapeutics Resistance and Associated lncRNAs in TNBC

A resistance to oncotherapeutics is a major challenge to efficacious patient management in the clinic. A resistance to therapy develops after prolonged exposure (acquired) or even before exposure (intrinsic) to cytotoxic and targeted therapies [57]. The development of drug resistance behaves similar to evolutionary processes, and the development of some of the resistance mechanisms are common between microbes and cancer cells [58,59,60]. There are multiple mechanisms of cancer drug resistance that include modification of the drug efflux, enhancing DNA damage repair, escape from apoptosis, immune system evasion, improvised and differential metabolisms, mutation of the drug targets, and epigenetic alterations [61]. The most common mechanisms of cancer drug resistance are (1) drug-dependent resistance, in which the efflux transporter is overexpressed, which enhances the efflux and reduces the uptake of the drug in a tumor cell; (2) target-dependent drug resistance that is affected by factors responsible for drug targets, such as deletion, translocation, amplification, and mutation; and (3) drug/target-independent drug resistance caused by drug target modification by an epigenetically or genetically altered cell signaling pathway [62,63,64].

The resistance existing before the patient is exposed to drugs is called innate or intrinsic resistance. The major causes of innate resistance are (1) heterogeneity of the tumor, including pre-existing insensitive subpopulations and associated cancer stem cells, (2) the activation of intrinsic pathways such as the glutathione (GSH)/glutathione S-transferase system [65], as well as the ABC transporter-mediated drug efflux [66], which can increase the drug metabolism or reduce the cellular drug accumulation, and (3) an inherent genetic mutation that can lead to a reduced sensitivity of tumors; for example, breast cancer gene 1 (BRCA1) signaling plays a critical role in the DNA damage response at the point of double-strand breaks (DSBs) via the homologous recombination pathway. The resistance of TNBC to DNA-damaging agents, including platinum and poly (ADP) ribose polymerase (PARP) inhibitors (PARPi), is because of germline mutations in BRCA1/2 [67].

An acquired resistance is due to (1) new mutations in a prolonged clinically treated tumor followed by regrowth of the tumor due to a newly emerged driver gene [68], (2) cross-talk that exists between the tumor cells and their microenvironment (modification of the tumor microenvironment can occur during the course of treatment), and (3) mutations or altered expression levels in a gene encoding target proteins. One example of acquired resistance is the tumor-initiating capacity and enhanced resistance to taxanes due to the CD49f+ cell population in TNBC, which increases during constant exposure to the drug and contributes to taxane resistance and tumor recurrence due to the “drug holiday effect”. In the absence of taxanes, the resilient CD49f+ population contracts, and taxane sensitivity is reestablished [69].

Some studies have indicated that lncRNAs are associated with the chemoresistance of cancer cells by impairing cellular responses as shown in Table 1 [70]. The overexpression of lncRNA H19 was associated with an acquired resistance to paclitaxel in TNBC patients, and the knockdown of H19 lncRNA led to the restoration of chemosensitivity in the paclitaxel resistance by enhancing the AKT signaling pathway [71]. The novel biomarker for adriamycin-resistant cells is ARA lncRNA, procured from an intron of the *PAK3* (p21-activated kinase 3) gene. The knockdown of ARA reduces breast and liver cancer cell proliferations and induces cell death, G2/M cell cycle arrest, and cell migration. Furthermore, ARA can regulate numerous signaling pathways, comprising metabolic pathways, the MAPK signaling pathway, cell cycle, and cell adhesion-related biological pathways, and modulate cellular processes, including protein-binding functions and transcriptional processes [72]. Thus, ARA might serve as a molecular biomarker for TNBC, as well as improve adriamycin-mediated chemosensitivity.

### 3.1. Modification of Drug Efflux

Multidrug resistance (MDR), by which cancer cells reduce the uptake or enhance the efflux of anticancer agents, is a significant and common occurrence and is a major obstruction to the success of cancer therapy. The common mechanism of drug efflux includes the overexpression of transmembrane transporters, primarily from the ABC transporter superfamily. This includes multidrug resistance-associated protein 2 (MRP2/ABCC2), P-glycoprotein (P-gp/ABCB1), and BC resistance protein (BCRP/ABCG2) [73]. The TNBC cell subline of TxR-HCC1806 (TxR-paclitaxel resistance) is characterized by MDR when exposed with paclitaxel and other anthracyclines and/or microtubule-binding agents (TBAs), leading to considerable enhancement (5.4-fold) of the paclitaxel efflux. The overexpression of MDR-1 and MRPs, along with an increased expression of ABC transporter proteins, resulted in the upregulation of antiapoptotic BCL-2 and the downregulation of proapoptotic protein Fas in TxR cells. Transcriptome analysis reveals that lncRNAs HIF1A(Hypoxia-inducible factor 1 alpha antisense RNA 2) and AK124454 are associated with TNBC and have an important role in invasion, metastasis, and multidrug resistance. Both lncRNAs are particularly related to G2-M arrest, which might be contributing to paclitaxel resistance in TNBC [74]. The MDR-associated lncRNA 00518 is upregulated in TNBC, and it enables MDR in BC by controlling the miR-199a/MRP1 axis. Linc00518 acts as a ceRNA of miR-199a to increase the MRP1 target gene expression. The knockdown of MRP1 in TNBC decreases the resistance of adriamycin, vincristine, and paclitaxel and induces apoptosis, while this effect could be eliminated by introducing the miR-199a inhibitor and linc00518 enhancer plasmids [75]. The overexpression of ABC transporters such as ABCC2 and ABCC3 can also contribute to multidrug resistance in TNBC [76].

### 3.2. Increasing DNA Damage Repair

Many chemotherapeutic drugs act to damage DNA, and affected cells exhibit a DNA damage response (DDR) to the drug, which may lead to drug resistance. For example, platinum drugs, alkylating drugs, topoisomerase inhibitors, and drugs such as 5-fluorouracil (5-FU) lead to increased DNA lesion repair to enhance drug resistance in cancer cells [77]. The expression of an upregulated lncRNA ANRIL is transcriptionally induced by DNA damage at the late stage of DDR and the indicator of a poor prognosis in TNBC. ANRIL acts as a molecular sponge and negatively regulates miR-199a to promote TNBC tumorigenesis. Therefore, miR-199a is a potential target for modulating the expression of ANRIL in TNBC [78,79]. Several lncRNAs such as mitotically associated lncRNA MANCR (Mitotically-associated long non-coding RNA) are responsible for decreased cell death with a concomitant increase in DNA damage in TNBC. The knockdown of MANCR leads to an increase in DDR and defective cytokinesis, suggesting that MANCR has a cytoprotective role in sustaining TNBC growth [80].

The alkylating agents such as temozolomide produce a DNA lesion of 24 to 30 bp by alkylation on guanine at the O6 position, but, in a process of direct reversion of DNA alkylation with the help of O-6-methylguanine-DNA methyltransferase (MGMT), damaged DNA can be restored without transfer of the damaged base [81]. DNA dioxygenases ABH2 (also known as ALKBH2) and ABH3 (also known as ALKBH3) modify 1-methyladenine and 3-methylcytosine back to adenine or cytosine, respectively [82]. Hence, the modulation of DDR affects the efficiency of chemotherapeutic drugs and may result in the accumulation of new mutations due to genomic instability, which may initiate de novo carcinogenesis [83].

### 3.3. Escape from Apoptosis

A resistance to various chemotherapeutics, such as 5-FU, doxorubicin, methotrexate, cyclophosphamide, and tubulin inhibitors, occurs in TNBC due to deregulation of the gene responsible for intrinsic apoptosis, such as TP53, CASP3, BCL-2, and BCL-xL [84,85,86,87]. hsa-miRNA-143-3p plays a vital role in drug resistance by regulating ant-apoptotic protein cytokine-induced apoptosis inhibitor 1 (CIAPIN1). The activation of MDR by the downregulation of miRNA-143-3p can upregulate the expression of its targeted protein CIAPIN1 in TNBC. The altered expression of hsa-miRNA-143-3p efficiently increases the sensitivity of drug-resistant TNBC cells to chemotherapeutics [88]. Onco-lncRNAs, such as SNHG12 (Small nucleolar RNA host gene 12), are upregulated in TNBC, promoting proliferation, migration, and inhibiting apoptosis. Transfection of the TNBC cell line with siRNA to the knockdown of SNHG12 results in increased rates of apoptosis. The transcription activation of SNHG12 is directly correlated with the binding of c-MYC to the promoter region of SNHG12 in TNBC. The expression pattern of matrix metalloproteinase 13 (MMP13) is directly proportional to the SNHG12 expression, and the degradation of MMP13 in TNBC promotes tumor metastasis and invasion. Therefore, MMP13 is a potential target of SNHG 12. It is reported that SNHG12 might act as a ceRNA to stabilize MMP13 or act as a scaffold-mediating RNA-binding protein in TNBC [89]. MALAT1 (Metastasis-associated lung adenocarcinoma transcript 1) and lnc00511 lncRNA are increased in expression in TNBC and can inhibit apoptosis, while the knockdown of MALAT1 promotes apoptosis in TNBC [90,91]. NEAT1(Nuclear enriched abundant transcript 1) is identified as a highly expressed lncRNA in TNBC. One study has examined the oncogenic role of NEAT1 in regulating apoptosis and cell regulation. The depletion of the expression of NEAT1 by using shNEAT1 sensitizes cells to chemotherapy, reduced the stemness and increases apoptosis in TNBC [71,92].Thus, modifications in the genes involved in apoptosis prevent chemotherapy-induced apoptosis, but modulation in regulatory non-coding RNAs leads to sensitizing chemo-resistant TNBC cells.

### 3.4. Immune System Evasion

Bou-Dargham et al. analyzed more than 1000 BC patients and observed that BC responds differently when treated with current immunotherapies such as immune checkpoint inhibitors and adoptive cell therapy, compared to other cancers [93]. Based on the differential expression of immune-related genes and apparent evasion mechanisms, they found that 77.4% of TNBC tumor specimens escape through transforming growth factor-beta (TGF-β), 48.0% through cytotoxic T-lymphocyte-associated protein 4 (CTLA4), 57.7% through decoy receptor 3 (DcR3), and 34.3% through programmed cell death-1 (PD-1). Due to their overexpression in BC, it is a prominent target for immunotherapy [93]. Novel lncRNA OSTN-AS1 (OSTN Antisense RNA 1), found by an integrated analysis of the ceRNA (competitive endogenous RNA) network, displays immunologic functions related to immune cell infiltration and immune-related marker expressions in TNBC and has a positive correlation with genes such as PDCD1, CTLA-4, and T-/B-cell receptor signaling involving CD-79 [94]. PD-1 blockade-resistant TNBC patients possess the upregulation of long intergenic non-coding RNA for kinase activation (LINK-A), which facilitates the K48-polyubiquitination-mediated degradation of the intrinsic tumor suppressor Rb, p53, and antigen peptide-loading complex (PLC). The knockdown of LINK-A stabilizes the Rb, p53 [95], and PLC components and sensitizes mammary gland tumors to immune checkpoint blockers. Therefore, the lncRNA-dependent downregulation of intrinsic tumor suppression and antigenicity provides a rationale for developing immunotherapy treatments [96]. Recent reports suggest that PD-L1 is robustly expressed in TNBC patient specimens. PD-L1 and PD-1 are crucial immunosuppressive molecules, and PD-L1 obstruction can considerably decrease T cell apoptosis and plays an important role in controlling T cell (Treg) maintenance and induction in tumor models [97]. Combinatorial approaches using immune checkpoint inhibitors, PD-1/PD-L1 antagonists, can be integrated with distinctly targeted therapies, including inhibitors of glucocorticoid-induced tumor necrosis factor receptor (GITR) and MAP2K, which has been proven advantageous in clinical trials for TNBC [98]. Currently, ipilimumab (anticytotoxic T lymphocyte-associated antigen 4 (anti-CTLA-4) monoclonal antibody) is under clinical trial for TNBC [99]. NKILA lncRNA has overexpressed in BC-specific CTLs and TH1 cells. NKILA regulates T cell sensitivity to activation-induced cell death by hindering the NF-κB activity. The knockdown of NFKILA with allocating CTLs inhibited the growth of BC and enhanced CTLs infiltration [100].

### 3.5. Changes in Metabolism

Oncometabolites such as succinate, fumarate, and D-2-hydroxyglutarate aberrantly accumulate in tumors and regulate certain epigenetic changes that are related to the malignant phenotype [101]. In BC cells, lactate and ketones are associated with augmented stemness and oxidative stress. Furthermore, glycine promotes rapid cell proliferation [102,103]. The expression of serine metabolism–associated enzymes such as phosphoglycerate dehydrogenase and phosphoserine phosphatase are highly expressed in TNBC [104].

The monocarboxylate transporter, which is responsible for lactate export, creates an acidic extracellular tumor environment that favors tumor progression by promoting migration and invasion [105]. In TNBC, the levels of glutamate, glutamine, and glutathione are increased, whereas proline, lysine, and valine are decreased in the presence of paclitaxel, which is indicative of an increased drug resistance in TNBC cells [106]. LncRNA H19 acts as a ceRNA to the sponge let-7, which upregulates Lin28 expression and may form a double-negative feedback loop in the maintenance of breast cancer stem cells. The depletion of H19 inhibits tumor growth in nude mice [107]. Drug-metabolizing enzymes (DME), such as cytochrome p450, which inactivate or activate the drug, are regulated by lncRNA H19 in TNBC. The conjugative class of enzymes, such as glutathione S-transferase, sulfotransferases and uridine diphosphate glucuronosyl-transferase, act on the drugs or xenobiotics and mediate their bioactivation. Among them, chondroitin sulfotransferase is a target of HOTAIR in TNBC. DME plays a crucial role in drug metabolism, elimination, and degradation [108,109,110]. The synthetic analog of glucose2-deoxy-D-glucose (2-DG) uptake by cells via the GLUT facilitative transporter is cytotoxic to cells and cannot be metabolized for energy. Cancer cells possess a higher rate of glycolysis compared to normal cells. Therefore, the effect of 2-DG with a combination of docetaxel or trastuzumab should be more selective for TNBC and should effectively starve TNBC cells [111]. This suggests that metabolomics associated with lncRNA offer a better understanding of drug resistance in TNBC.

## 4. Therapeutic Strategies to Target lncRNAs

LncRNAs may be preferred therapeutic targets because of their restricted spatiotemporal expressions and ability to modulate the expression or function of interacting partners [116]. LncRNAs have been successfully targeted by employing several approaches: (1) transcriptional block, (2) transcript degradation, (3) block interaction, and (4) functional inhibition.

### 4.1. CRISPR-Cas9

Growing evidence suggests that lncRNA can be targeted using the CRISPR-Cas9 genome-editing approach to block the transcription of lncRNA. Recently, high-throughput genome deletion using paired guide RNA has been successfully used for identifying the lncRNAs responsible for the growth and survival of cancer cells [117]. The CRISPR-Cas9 technique allows us to delete the genome at a precise location with a specific size and high fidelity. Several lnc*RNA*s, such as lncRNA RoR, lncRNA BC200, lncRNA AK023948 (breast carcinoma), lncRNA UCA (Urothelial cancer associated 1) (bladder), lncRNA 01116 (prostrate), lncRNA CCAT2 (colon cancer-associated transcript 2), lncRNA 21A (colorectal carcinoma), and lncRNA PANDAR (gastric cancer), have been successfully edited by CRISPR-Cas9, and functional validation experiments have proved that these lncRNAs are oncogenic in nature [118]. Moreover, the development of nonviral delivery approaches for CRISPR-Cas9 genome editing has provided an attractive method for therapeutic use.

### 4.2. Antisense Oligonucleotides (ASOs)

ASOs are also a most powerful strategy for targeting lncRNAs. They have been approved by the FDA for the treatment of hypercholesterolemia and transthyretin amyloidosis. Recently, MALAT1 ASOs have been reported to reduce tumor growth in a preclinical mouse model for BC [119]. Targeting lncRNA PVT1(Plasmacytoma variant translocation 1) by locked nucleic acid (LNA) rendered the ovarian cancer cells sensitive to cisplatin [120]. Similarly, using LNAs against lncRNA ARSR sensitized cancer cells to sunitinib [121]. Additionally, LNAs targeting hypoxia-inducible factor (HIF) 1a (NCT00466583), the Bcl-2 oncoprotein [122], and the androgen receptor are in clinical trials. Gas5 (growth arrest-specific 5) was shown to be downregulated in TNBC, and LNAs mimicking its binding site on hormone receptors in TNBC cells induced apoptosis [123].

### 4.3. Small Interfering RNA (siRNA)/Short Hairpin RNA Mediated the Silencing of lncRNAs

siRNA/shRNAs degrade their target through an RNA-induced silencing complex in association with argonaut 2. LncRNAs are easily targeted by employing a siRNA approach that allows an understanding of the biological relevance of the lncRNAs in human malignancies. For example, MALAT1-specific siRNA displayed a significant decrease in tumor growth, metastasis, invasion, and cell cycle arrest [124]. HOTAIR siRNA has shown a reduced matrix invasion in breast carcinoma cells [125]. Moreover, the shRNA-mediated knockdown of HOTAIR in gastric cancer cells decreased the tumor formation and metastatic potential in nude mice [126]. Therefore, siRNA/shRNA against lncRNAs may be used as a therapeutic approach.

### 4.4. Small Molecule Inhibitors

Numerous studies have reported that lncRNAs have strong binding affinities with different components inside the cell. Small molecule inhibitors, which can hamper the lncRNA recognition site to further interact with a binding partner, may inhibit the effects of lncRNA in disease. The small-molecule inhibitor that intrudes on the folding of RNA into the secondary or tertiary structures can be used as a target to inhibit the functions of lncRNA [127]. The upregulation of HOTAIR is responsible for TNBC migration and proliferation. A recent study demonstrates the effective blocking of the HOTAIR: PRC2 interaction, by using a peptide nucleic acid (PNA)-based approach to inhibit the invasion and increase the chemotherapeutic sensitivity in TNBC. To deliver anti-lncRNA into the tumor microenvironment, PNA was conjugated with a pH-low insertion peptide [128]. Further, Fatema et al. orchestrated an alpha screening in search of the HOTAIR: PRC2 complex and brain-derived neurotrophic factor antisense (BDNF-AS) small molecular inhibitors. Though ellipticine was identified, this in vitro study depicts the ability of small molecules to modulate the lncRNA protein [129]. Moreover, the integration of an antagonist against HOTAIR leads to competitive inhibitions with PRC2 and LSD1, which can inhibit HOTAIR and reduce metastasis in BC [130].

### 4.5. Ribozymes or Deoxy Ribozymes

Hammerhead ribozymes are catalytic RNAs with a high target specificity. Hammerhead ribozymes that specifically target one lncRNA at a time can be used to block oncogenic lncRNAs in cancer. Pavco and colleagues have used ribozymes specifically targeting vascular endothelial growth factor receptor (VEGFR) in colon cancer. Their data showed that the anti-VEGFR [131] ribozyme suppressed tumor growth, as well as liver metastasis [70]. This suggests that ribozymes targeting lncRNAs may be potential therapeutic molecules for the treatment of human malignancies [126].

### 4.6. Natural Antisense Transcripts

Natural antisense transcripts (NATs) are present in a similar way to lncRNAs. Therefore, it will be worthwhile to target the NATs that will eventually increase the expression of sense mRNA. This strategy will be very useful to compensate for the effects of tumor-suppressive lncRNAs [126,128,132]. The NATs such as PDCD4-antisense RNA1 (PDCD4-AS1) are responsible for TNBC progression, which positively regulates the tumor suppressor, sense protein-coding partner PDCD4 (programmed cell death 4) expression in a cis manner. Both PDCD4-AS1 and PDCD4 show reduced expressions in TNBC patients; PDCD4-AS1 acts upstream to stabilize PDCD4 RNA by forming a RNA duplex and controlling the expression between PDCD4 RNA, HuR (decay factor), and AUF1 (stabilizing factor). This demonstrates that NAT lncRNA regulates the expression of a key tumor suppressor sense partner in TNBC progression [133]. Recently, the OPKO CURNA biotech-based enterprise from the USA successfully synthesized oligonucleotides targeting the NAT function by steric hindrance. In addition, another biotech firm, RaNa, from the USA, also successfully targeted the NAT interaction using PRC2 oligonucleotides [134].

## 5. Conclusions and Future Perspectives

Chemotherapy and FDA-approved molecular-targeted therapies are treatment methods used therapeutically to prolong patient survival. However, drug resistance is a major challenge hindering the efficacious treatment of TNBC. During the last decade, significant efforts have been dedicated to the exploration of mechanisms of drug resistance in human malignancies and, also, approaches to resensitize cancer cells to specific therapeutics. Many protein-coding genes such as *ABCG2*, *MDR1*, *MRP*, and their regulatory lncRNAs play a critical role in chemoresistance and might be used as targeted therapeutics against cancerous cells [135]. LncRNAs are ideal biomarkers that are crucial in quasi-personalized treatment selection and, subsequently, in the enhancement of the consequent prognosis [17]. Moreover, lncRNAs have been reported to act as TNBC-suppressor genes or oncogenes that can increase or reduce the resistance of TNBC to treatments, including cisplatin, tamoxifen, docetaxel, and 5-FU [136]. Hence, there is an exciting opportunity to develop lncRNA-based cancer therapies that can be applied in clinical practice. While research on lncRNAs and chemotherapeutic resistance remain in the early stages, lncRNAs may be potential candidates for developing novel strategies to resensitize cancer cells to chemo or targeted therapies. Consequently, more work is required to identify lncRNAs involved in drug resistance in TNBC and to decipher their functions and the mechanisms utilized.

## Figures and Tables

**Table 1:** Details of triple-negative breast cancer (TNBC)-associated long non-coding (lnc)RNAs and their roles in chemoresistance in TNBC are given in the table.

lncRNAs	Targets	Mechanisms	Functions	Drugs	Expression Patterns	Restore the Expression Pattern of lncRNAs	References
BORG	NF-ĸB signaling, RPA1	The BORG reveal its strong chemo-resistant activities and induction and activation of the NF-κB pathway; moreover, activates BORG expression in a doxorubicin-mediated feed-forward loop	Metastasis, poor prognosis, and chemoresistance	Doxorubicin	Upregulated	CRISPER/Cas9 facilitated inhibition of BORG expression and restored chemosensitivity and apoptosis in TNBC	Alex J. Gooding et al. [112]
HCP5	Regulating PTEN expression	Downregulation of PTEN expression and upregulates p-AKT expression	Cisplatin-resistance	Cisplatin	Downregulation	Overexpression of HCP5 upregulated the expression of PTEN led to reestablish the function of DNA repair and drug sensitivity in TNBC, and down-regulated the expression of p-AKT.	Jingjing Wu et al. [113]
HIF1A-AS2	Integrated mRNA-lncRNA signature	N/A	Cell proliferation, invasion, and chemoresistance	paclitaxel	N/A	N/A	Yi-zhou jiyang et al. [74]
AK124454	Integrated mRNA-lncRNA signature	N/A	Cell proliferation, invasion, and chemoresistance	paclitaxel	N/A	N/A	Yi-zhou jiyang et al. [74]
H19	AKT Signaling Pathway	Regulates the AKT Signaling pathway	Cell proliferation and chemoresistance	paclitaxel	upregulation	Knockdown regulation of H19 restores chemosensitivity in paclitaxel resistance TNBC by modulating the AKT signaling pathway by triggering apoptosis	Jiguang Han et al. [71]
NEAT1	N/A	N/A	NEAT1 has been implicated in cell growth, migration, and invasion and regulated miR-488, ZEB1, and chemoresistance	Synergistic and combinational drugs (cisplatin and taxol)	Upregulation	Knocking down the expression of NEAT1 led to sensitized cells to chemotherapy and reduced stemness in TNBC	Vivian Yvonne Shin et al. [92]
lincRNA-RoR	microRNA-145	lincRNA-RoR in TNBC function as ceRNA and modulating miRNA-145 via targeting the ARF6 pathway	Drug Resistance and Metastasis	5-fluorouracil (FU) resistance via miR145	Upregulated	Knockdown lincRNA-ROR, restore miR-145, and regulate cell invasion	Gabriel Eades et al., Rui-Lei Liu et al. [114,115]
